# Invalid and Crippled Children

**Published:** 1920-11-06

**Authors:** 


### Invalid and Crippled Children.

A JOINT CONFERENCE on "The Care of Invalid and Crippled Children" is to be held by the Invalid Children's Aid Association and the Central Committee for the Care of Cripples on Tuesday and Wednesday, November 16 and 17, in the Council Chamber of the Guildhall, London. The Lord Mayor will open the Conference, and the Right Hon. H. A. L. Fisher, President of the Board of Education, will give an address. The chair will be taken at the four sessions by Sir William Bennett, K.C.V.O., F.R.C.S., the Hon. Sir Arthur Stanley, G.B.E., C.B., M.V.O., Sir Robert Jones, K.B.E., F.R.C.S., and Sir Napier Burnett, K.B.E., F.R.C.S. The subjects for discussion include "The Bearing of Recent Legislation on the Work for Physically Defective Children," "Co-operation between the State and Voluntary Bodies," "Accounts of Existing Institutions for the Treatment and Education of these Children in the United Kingdom, in America, and on the Continent," "The Treatment of Heart Cases," and "The Scheme for Future Development suggested by the Central Committee for the Care of Cripples." Among the speakers will be Captain Warburg, O.B.E., Chairman Public Health Committee L.C.C., Dr. Hope (Liverpool), Dr. Robertson (Birmingham), Miss Adler, J.P., L.C.C., Dr. Wheatley (Shropshire), Mr. Woollcombe (C.O.S.), Mr. Platt (Manchester), and Sir Robert Jones. The speeches will be followed by discussion.

The Central Committee for the Care of Cripples has collected a considerable amount of information as to the numbers of physically defective children throughout the country and as to the amount of accommodation available for them at the present time, and the Conference should be of real importance in determining the direction of future work.

Tickets of membership admitting to all sessions of the Conference (price 5s.) may be obtained from the honorary secretaries, Mrs. Munro, I.C.A.A., Denison House, Vauxhall Bridge Road, S.W., or Mrs. Townsend, C.C. for C.C., 20 Berkeley Street, W. 1, together with all further particulars. The sessions commence at 10.30 and 2.30 on each day. Visits to various typical institutions, such as Queen Mary's Hospital, Carshalton, St. Vincent's Home, Pinner, and to L.C.C. Open-air and Physically Defective Schools, are arranged for November 15 and 18 by motor char-à-banc from Denison House; tickets, 3s. 6d. return to Carshalton or Pinner, and 2s. 6d. to the Schools.

